# Evaluating the Risk of Comorbidity Onset in Elderly Patients After a Cancer Diagnosis

**DOI:** 10.21203/rs.3.rs-5189676/v1

**Published:** 2024-12-16

**Authors:** Tamzid Islam, Saiful Islam Saif, Naima Alam, Sam Pepper, Isuru Ratnayake, Dinesh Pal Mudaranthakam

**Affiliations:** University of Kansas Medical Center; University of Kansas Medical Center; University of Kansas Medical Center; University of Kansas Medical Center; University of Kansas Medical Center; University of Kansas Medical Center

**Keywords:** Cancer treatment, Treatment Side-effects, Comorbidity development, Propensity Score Matching

## Abstract

**Background:**

Cancer is a critical disease that affects a person physically, mentally, socially, and in many other aspects. During the treatment stage of cancer, patients suffer from various health complexities, especially elderly people, which might result in the onset of other diseases development of a comorbid condition. Several studies have shown comorbidity plays a crucial role in cancer survival. However, there remains a lack of comprehensive statistical techniques at the national level studies to assess the significance of comorbidities development in cancer. Our research aims to address this gap by comparing cancer and non-cancer individuals over four years’ time period.

**Methods:**

The Health Retirement Study (HRS) data was used to extract information from 6651 participants aged more than 50. Within a 4-year time span, cross-sectional observations were created whether comorbidities or not based on the development of diseases such as high blood pressure, diabetes, heart disease, stroke, lung disease, and psychological disease.

**Results:**

The multivariable logistic regression model, we observe higher chances of developing comorbidity (OR=1.321, p-value 0.0051) among the cancer group compared to the non-cancer group, adjusting the socio-economic factors. Moreover, the socio-economic factors were found to be significantly associated with cancer leading to applying the propensity score matching with (1:3 matching). Finally, the balanced data also showed significantly higher chances of developing comorbidity (OR=1.294, p-value 0.0207) among cancer patients.

**Conclusions:**

The above findings demonstrated the imperative development of enhanced treatment protocols, which prioritize the overall health of cancer patients, thereby reducing their susceptibility to additional illnesses.

## Introduction

Cancer remains a predominant global health challenge in the 21st century [1]. According to the American Cancer Society, approximately one in five individuals develop cancer in their lifetime, with around one in nine men and one in twelve women facing death due to the disease [2]. In the United States alone, more than 2 million new cancer cases and over half a million deaths are projected in 2024 [3]. This staggering statistic underscores the urgent need for enhanced research and innovative treatments. The cancer-related studies describe how this disease affects the healthy tissues of a patient and causes physical complications leading to death[4–6]. Some studies also demonstrated an increase in long-term survivorship due to current improvements in cancer treatment despite some long-term effects [7, 8]. Beyond the physical toll, cancer profoundly impacts mental, social, and financial well-being [9–11]. However, cancer treatment procedures like chemotherapy can cause the immunity of a cancer patient to be compromised [12]. This vulnerability is especially pronounced in elderly cancer patients, who already face numerous health challenges, thus increasing their risk of developing comorbidities [13].

A major aspect of cancer’s complexity is the frequent presence of comorbidities among cancer patients[14]. Comorbidities such as cardiovascular diseases, diabetes, and hypertension are prevalent among cancer patients and can lead to complications. Previous statistics indicates that over 30% of cancer patients aged 65 and older have multiple chronic conditions [15, 16]. The presence of comorbidities is associated with increased psychological distress, decreased life, and an elevated risk of functional decline[17, 18]. The circumstances get worse when these physical and mental conditions result in work impairment, leading to financial hardship not only for the patient but also for the whole family [19]. Cancer patients with comorbidities frequently reported to have higher levels of anxiety and depression, which can further hinder their ability to manage the demands of cancer treatment [17][20]. Moreover, physical limitations caused by commorbidites can restrict their ability to udergo necessary therapies such as chemotherapy or surgical interventions[21, 22]. Previous research indicated that patients with severe comorbidities are less likely to reveive standard treatments which often conclude in delayed diagnoses[23, 24]. These delay can obscure cancer symptoms and leads to later-stage diagnoses that are linked with poorer prognosis[25, 26]. It highlights the importance of focusing on the development of comorbidities when evaluating cancer patients as those can worsen the burden of the patients.

Previous research has consistently shown high rates of comorbidity among cancer patients, more specifically in the elderly age group [20, 27]. The development of comorbidities in cancer patients can be influenced by various socio-economic factors, including race, age, and sex, as these factors can affect susceptibility to different diseases. However, past studies often fail to adjust the conclusion considering these confounding effects of socio-demographic factors. In this study, we aim to fill this gap by assessing the occurrence of comorbidity among an elderly age group after they are diagnosed with cancer, addressing the combined confounding effect of other factors. By applying statistical techniques, including logistic regression models, we aim to draw valid conclusions regarding the influence of comorbidities on a nationally representative sample of cancer and non-cancer patients. This research seeks to contribute to advancing healthcare treatment strategies better to address the complexities of cancer and its related conditions.

## Materials and Methods

### Data and Variables:

The study protocol was approved by the Institutional Review Board (IRB) at the University of Kansas Medical Center under the study number STUDY00147028. Access to the restricted data from Health and Retirement Study (HRS) at the University of Michigan (Ann Arbor, MI) was approved following the review and approval procedures of HRS team. The HRS was approved by the institutional Reviewing Board at the University of Mchigan and the National Institute on Aging (HUM0061128). All participants filled in the informed consent forms.

**Figure 1:**
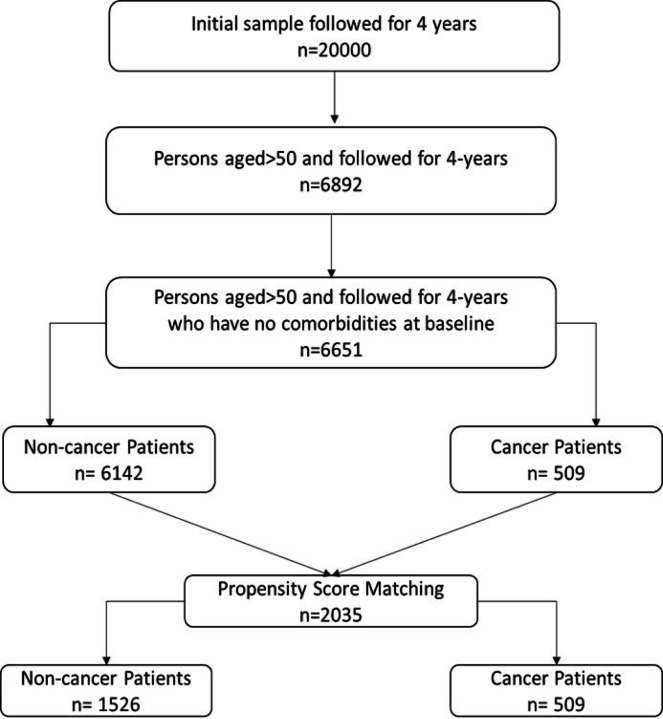
Consort Diagram of inclusion criteria and study participants after propensity score matching

To investigate comorobidity development contributing to worsening health outcomes among cancer patients’ and our study utilized the restricted dataset of the University of Mihigan’s Health and Retirement Study. The HRS conducted by the Institute of Social Research (ISR) at the University of Michigan is a national longitudinal study focusing on the economic, health, marital and family status of older Americans as well as their public and private support systems. This survey included 20,000 individuals aged 50 or older in America and information was collected in every two years from 2002 to 2016. Our study’s inclusion criteria required participants to have no reported comorbidities at the baseline or initial wave. This allowed us to observe the development of comorbidities over time, specifically over a 2 wave (4 years) period which provided a longitudinal perspective on health outcomes. The initial sample had approximately 20000 individuals. Based on inclusion criteria of no comorbidities at the initial wave, the sample comprised 6,651 individuals, including 6,142 non-cancer and 509 cancer patients. Figure 1 illustrates the data cleaning process.

The primary outcome variable, Comorbidity, was classified as ‘No’ (absence of comorbidities) and ‘Yes’ (presence of ≥ 1 comorbidity), which includes high blood pressure (BP), diabetes, lung disease, heart disease, stroke, and psychological problems. The exposure variable of interest, Cancer, was classified as ‘No’ and ‘Yes’. A range of covariates was also included to comprehensively adjust for potential confounders. These encompassed Gender (Male, Female), Hispanic status (No, Yes), Race (White/Caucasian, Black/African American, Other), Educational Attainment (Below College Degree, College Degree and above), Perceived Health Change (Somewhat Better, Same, Somewhat Worse), Depressed status (No, Yes), Smoking history (No, Yes), Poverty status (Above Poverty, Below Poverty), Body Mass Index (BMI) category (Normal, Obese, Overweight, Underweight), Comorbidities (No, Yes), and Income.

### Statistical Analysis

#### Bivariate Analysis

Bivariate analysis was conducted as the first step in our investigation. This involved analyzing the association between cancer status and covariates such as race, Hispanic status, BMI, and smoking status, among others. We examined each covariate for its relationship with the cancer status to identify potential effects. For categorical covariates, we utilized the chi-square test to assess associations, while for continuous covariates, we used a t-test to compare their means.

#### Propensity Score Matching

We used propensity score matching (PSM) to address the observed associations and potential confounders identified in the bivariate analysis [21]. The primary goal of PSM was to reduce confounding bias and adjust for the baseline characteristics between cancer and non-cancer cohorts. This approach enhances the validity of causal inference drawn from the observational data. A Greedy matching algorithm was used for its efficacy in pairing individuals from the treatment and control groups based on their propensity scores (PS), which were estimated through logistic regression models. The matching was executed at a 1:3 ratio to ensure each cancer patient was matched with three non-cancer patients. This optimizes the statistical power while maintaining the matching quality. A caliper width of 0.25 standard deviations of the logit of the propensity score was specified. Following PSM, the matched sample consisted of 509 cancer patients and 1,526 non-cancer patients. The p-value for the controlled criteria (factors included in the PSM) was tested to see if they are significant or not.

#### Logistic Regression Analysis

The final stage of the analysis involved logistic regression modeling to evaluate the impact of cancer on the development of comorbidities. Comorbidity development was our dependent variable, and cancer status was the main factor we were looking at. We also included other important factors identified earlier in the bivariate. This multivariate approach allowed us to see various potential influences, allowing us to highlight the role of cancer that might play a crucial role in the development of other health conditions more clearly. The results are presented in terms of odds ratio (OR). For each OR, we also looked at the p-values and confidence intervals to determine whether the association between cancer and comorbidity development is statistically significant or not. R studio version 4.3.0 and SAS software version 9.4 were used to conduct our analysis.

## Results

### Participant Characteristics

Among the total sample of 6892 individuals, 6142 (92.3%) participants had no cancer, while 509 (7.7%) were reported as cancer-affected. Gender distribution indicated that 509 participants (7.7%) were male, whereas the majority comprised females with 3892 (58.5%) individuals. In terms of Racial demographic 5248 (78.9%) identified as White/ Caucasian, 878 (13.2%) as Black/ African American, and 806 (12.1%) belonged to other racial categories. Notably, the majority of participants were reported as being above the poverty line, a balanced ratio observed in the history of smoking, and a small number of people reported underweight BMI.

### Comorbidity and Cancer Status Distribution

**Figure 2:**
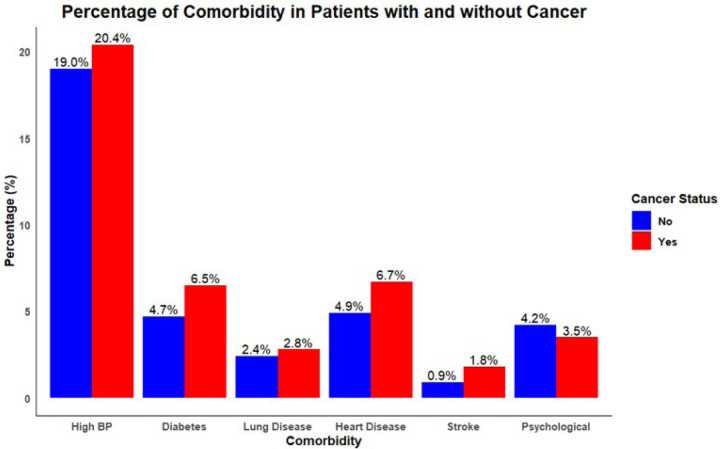
Distribution of Comorbidities by type among Cancer vs non-cancer groups

**Figure 3:**
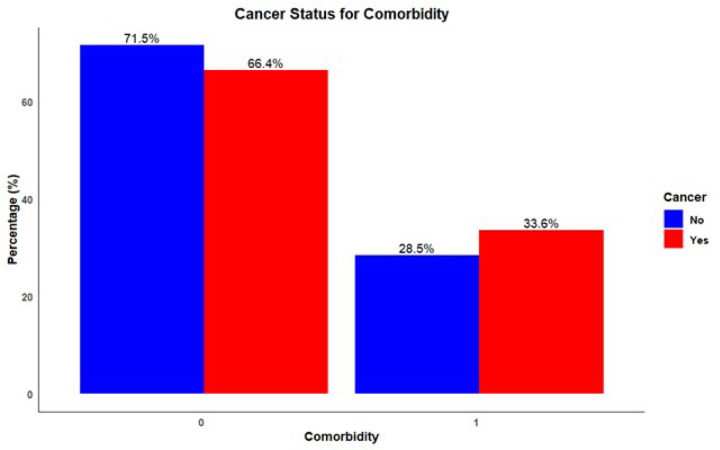
Distribution of Comorbidities among Cancer vs non-cancer groups

Distribution of various comorbidities among individuals with and without a cancer diagnosis revealed high blood pressure (BP) was the most prevalent comorbidity in both groups, with 19.0% of non-cancer patients and 20.4% of cancer patients affected (Fig. 2). Diabetes was the next most common condition among non-cancer individuals at 4.7%, while it was slightly more prevalent in cancer patients at 6.5%. Similarly, lung disease, heart disease, and stroke demonstrated a higher prevalence in cancer patients compared to non-cancer participants. Regarding the overall presence of any comorbidity, 71.5% of individuals without cancer did not report any comorbidities, while 66.4% of cancer patients were free from additional reported conditions (Fig. 3). Notably, the presence of at least one comorbidity was reported by 28.5% of non-cancer patients and by a notably higher proportion, 33.6% of cancer patients.

### Association Between Comorbidity and Cancer Status along with Socio-Economic Factors

Bivariate analyses between the development of comorbidity and various covariates were summarized in (Table 1). Utilizing Chi-square tests, statistically significant associations (p < 0.05) were found between the development of comorbidities and cancer status, race, highest degree attained, BMI, poverty status, and smoking history. Furthermore, bivariate analyses between cancer and non-cancer groups and other covariates demonstrated significant associations (Supplementary Table 01). Significant associations (p < 0.05) were observed in cancer versus non-cancer groups with race, Hispanic ethnicity, BMI proxy, poverty status, and smoking history based on chi-square tests.

### Adjusted and Unadjusted Effects of Cancer Status on Comorbidity Development

Univariable and Multivariable logistic analyses were conducted to assess the adjusted and unadjusted effects of socio-economic factors on the development of comorbidity for cancer and non-cancer group. Analysis of the unadjusted logistic model revealed cancer status as a significant predictor of the development of comorbidities, whereas participants in cancer group were associated with a 28.7% increase in the odds of comorbidity development compared to non-cancer individuals (OR: 1.287, 95% CI: 1.038, 1.595) (Supplementary Table 02). After adjusting for potential cofounders by multivariable logistic regression, the cancer group was still significantly associated with development comorbidities, while the likelihood of developing comorbid conditions in cancer patients increased to 32.1% (OR: 1.321, 95% CI: 1.087, 1.605) compared to non-cancer respondents (Supplementary Table 02). Notably, for the adjusted model, covariates such as gender, race, and Hispanic ethnicity did not exhibit a statistically significant effect on the development of comorbidity. However, significant impacts were found on covariates such as BMI, poverty, smoking, and educational status. Specifically, it was observed that individuals with a college degree and above had a reduced likelihood of comorbidity compared to individuals without education (OR: 0.792, 95% CI: 0.702, 0.893). Obesity was a predictor of comorbidity development with an 80.3% increase in odds (OR: 1.803, 95% CI: 1.561, 2.082), while being overweight was also associated with a higher odds of comorbidities compared to BMI with normal weight. Moreover, individuals with below poverty and a history of smoking were significantly associated with higher odds of developing comorbid conditions.

### Post-Propensity Score Matching Assessment

The balance of covariates post-propensity score matching was conducted based on cancer and non-cancer groups to ensure that the matched groups were comparable with demographic and socio-economic factors (Table 2). Multivariable logistic analyses based on post-propensity score matching data revealed no significant differences between cancer and non-cancer groups for covariates such as gender, race, Hispanic ethnicity, educational attainment, BMI, and smoking status. These non-significant reflects the effectiveness of the matching process in achieving balanced across examined covariates.

### Adjusted and Unadjusted Effects of Cancer Status on Comorbidity Development after balancing data

Propensity score adjusted and unadjusted effects of cancer on the development of comorbidities were obtained from a univariable and multivariable logistic regression model (Table 3). The unadjusted model on balancing data revealed presence of cancer was significantly associated with a 28.7% increase in odds of comorbidity development (OR: 1.287, 95% CI: 1.038, 1.595). After adjusting for covariates on balanced data, cancer group was still significantly associated with increased odds of combordities (Table 3). Notably, covariates including gender, race, Hispanic and smoking status did not exhibit significant associations. However, covariates including educational attainment, BMI, poverty was significantly associated with comorbidities development.

## Discussion

Our study highlighted the significant association between cancer diagnosis and increased likelihood of developing comorbidities in elderly patients. All comorbidities available in our study cohort, including high blood pressure (BP), diabetes, lung disease, heart disease, stroke, and psychological problems, had a higher percentage in cancer patients than in healthy individuals. Previous research suggested that hypertension is one of the most frequent comorbidities in cancer and can increase the risk of mortality [14, 22]. Diabetes is another leading comorbidity in cancer patients that can affect both treatment outcomes and overall survival [23, 24]. Chronic lung disease are common among cancer patients, particularly those with lung cancer, and it’s a common comorbidity in cancer, which may lead to increased hospitalizations and decreased quality of life [17] [25]. Heart disease and stroke are also critical comorbidities that can complicate cancer conditions and increase the risk of mortality [26–29]. Cancer affects not only physical but also psychological and social well-being, which can further complicate the management of comorbidities [30].

Our study emphasizes a substantial public health concern, considering the aging population and the rising incidence of cancer [31–36]. Elderly cancer patients have an increased risk of developing comorbidities due to the interplay of aging, cancer treatment, and side effects [37–40]. Previous research indicated that older cancer survivors have an increased chance of mortality mainly due to new comorbidities in post-diagnosis [41]. The stress and anxiety associated with a cancer diagnosis can worsen existing health conditions and may contribute to the onset of new ones, particularly in elder patients. All these findings demonstrated an urgent need for health strategies to reduce the chance of developing comorbidities in elderly patients.

Our study’s use of propensity score matching (PSM) is a strong methodological choice that enhances the validity of the findings by controlling for confounding variables [42]. PSM enabled a more precise comparison between cancer and non-cancer groups by balancing based on demographic factors, which is particularly important given the disparities in health outcomes among different populations. [29] Similar to our study, previous studies employed PSM that demonstrate it’s effectiveness in estimating treatment effects in heterogeneous populations[43]. Furthermore, socio-economic factors, including BMI, poverty status, smoking status, and educational status, were found to be significantly associated with comorbidity development in unmatched data. However, after applying PSM, educational attainment, BMI, and poverty remained significant in matched data. Our previous research suggested that cancer patients living below the poverty line have an increased risk of mortality[10]. One of the limitations of this study is our cohort included several types of cancer. Although this approach allows for a boarder understanding of comorbidity prevalence across cancer patients, it might overlook the comorbidity patterns of each specific cancer type.

## Conclusion

By taking a comprehensive and personalized approach, involving a multidisciplinary team, and focusing on preventive care and lifestyle management, cancer treatment plans can be tailored to minimize the onset and exacerbation of comorbidities. These strategies prioritize the patient’s overall health and quality of life, ensuring both the cancer and comorbidities are managed effectively.

## Supplementary Material

Supplement 1

## Figures and Tables

**Table 1:** Bivariate Distribution between development of comorbidity vs other covariates

Characteristic	Overall N = 6,892	No of Comorbidities developed		p-value*
		0 N = 4,899	≥ 1 N = 1,993	
**Cancer**				0.014
No (0)	6,142.0 (92.3%)	4,394.0 (92.9%)	1,748.0 (91.1%)	
Yes (1)	509.0 (7.7%)	338.0 (7.1%)	171.0 (8.9%)	
**Gender**				0.500
Male (1)	2,759.0 (41.5%)	1,976.0 (41.8%)	783.0 (40.8%)	
Female (2)	3,892.0 (58.5%)	2,756.0 (58.2%)	1,136.0 (59.2%)	
**Race-masked**				0.020
White/Caucasian (1)	5,248.0 (78.9%)	3,767.0 (79.6%)	1,481.0 (77.2%)	
Black/African American (2)	878.0 (13.2%)	590.0 (12.5%)	288.0 (15.0%)	
Other (3)	525.0 (7.9%)	375.0 (7.9%)	150.0 (7.8%)	
**Hispanic**				0.200
No (0)	5,845.0 (87.9%)	4,173.0 (88.2%)	1,672.0 (87.1%)	
Yes (1)	806.0 (12.1%)	559.0 (11.8%)	247.0 (12.9%)	
**Highest degree**				< 0.001
Below College Degree (0)	4,426.0 (66.5%)	3,053.0 (64.5%)	1,373.0 (71.5%)	
College Degree and Above (1)	2,225.0 (33.5%)	1,679.0 (35.5%)	546.0 (28.5%)	
**BMI Proxy**				< 0.001
Normal	2,501.0 (37.6%)	1,895.0 (40.0%)	606.0 (31.6%)	
Obese	1,443.0 (21.7%)	911.0 (19.3%)	532.0 (27.7%)	
Overweight	2,645.0 (39.8%)	1,884.0 (39.8%)	761.0 (39.7%)	
Underweight	62.0 (0.9%)	42.0 (0.9%)	20.0 (1.0%)	
**Whether in Poverty**				< 0.001
Above Poverty (0)	5,697.0 (85.7%)	4,103.0 (86.7%)	1,594.0 (83.1%)	
Below Poverty (1)	954.0 (14.3%)	629.0 (13.3%)	325.0 (16.9%)	
**Smoke Ever**				< 0.001
No (0)	3,110.0 (46.8%)	2,294.0 (48.5%)	816.0 (42.5%)	
Yes (1)	3,541.0 (53.2%)	2,438.0 (51.5%)	1,103.0 (57.5%)	

Note:

* chi-square test

**Table 2:** Assessment of covariate balance for cancer based on matched data

Analysis of Maximum Likelihood Estimates		
Parameters	Odds Ratio (95% Confidence Interval)	p-value
Gender (Female)	1.034 (0.834,1.283)	0.759
Race (White/Caucasian)	0.996 (0.546,1.817)	0.990
Race (Black/African American)	1.021 (0.51,2.042)	0.954
Hispanic (Yes)	0.996 (0.668,1.484)	0.984
**Highest degree (College Degree and Aboce)**	1.007 (0.811,1.249)	0.952
**BMI (Obese)**	0.976 (0.724,1.316)	0.873
BMI (Overweight)	0.990 (0.789,1.242)	0.930
BMI (Underweight)	0.979 (0.409,2.345)	0.963
Whether in Poverty (Below Poverty)	1.057 (0.752,1.484)	0.751
**Smoke ever (Yes)**	1.000 (0.812,1.231)	0.999

**Table 3:** Propensity score–adjusted effects of covariates on development of comorbidity obtained from logistic regression model

Analysis of Maximum Likelihood Estimates				
Parameter	Unadjusted Model		Adjusted	
	Odds Ratio (95% Confidence Interval)	p-value	Odds Ratio (95% Confidence Interval)	p-value
**Cancer (Yes)**	1.287(1.038,1.595)	0.022	1.294 (1.04,1.609)	0.021
Gender (Female)			1.023 (0.831,1.258)	0.832
Race (White/Caucasian)			1.037 (0.58,1.853)	0.904
Race (Black/African American)			1.107 (0.571,2.146)	0.764
Hispanic (Yes)			1.069 (0.736,1.552)	0.728
**Highest degree (College Degree and Aboce)**			0.708 (0.571,0.877)	0.002
**BMI (Obese)**			1.804 (1.368,2.377)	< 0.001
BMI (Overweight)			1.225 (0.98,1.53)	0.074
BMI (Underweight)			1.437 (0.645,3.198)	0.375
Whether in Poverty (Below Poverty)			1.615 (1.188,2.197)	0.002
**Smoke ever (Yes)**			1.099 (0.899,1.344)	0.355

## Data Availability

The data used to conduct this research was a public data set provided by the University of Michigan Health Retirement Study team. Data can be accessed after appropriate approval is obtained (for more information, please follow the link: https://hrsdata.isr.umich.edu/data-products/public-survey-data?_gl=1*u7yv15*_ga*MjA1ODQ2Mzk2Ni4xNzEzODkxNDkz*_ga_FF28MW3MW2*MTcyNzEwNjgwNS4zLjEuMTcyNzEwNjgyNC4wLjAuMA). The data product utilized for our study is labeled as Biennial Data 2018 HRS Core under the Public Survey Data.
