# Supplementary material for: Evaluating the Risk of Comorbidity Onset in Elderly Patients After a Cancer Diagnosis
Source: Res Sq. 2024 Dec 16:rs.3.rs-5189676. Preprint. [Version 1] doi: 10.21203/rs.3.rs-5189676/v1 (PMC11702800; doi:10.21203/rs.3.rs-5189676/v1)
Supplement: Supplement 1 [file NIHPPRS5189676v1-supplement-1.pdf]

Supplementary Files

This is a list of supplementary files associated with this preprint. Click to download.

- SupplementaryMaterials.docx
